# Challenges in mapping European rare disease databases, relevant for ML-based screening technologies in terms of organizational, FAIR and legal principles: scoping review

**DOI:** 10.3389/fpubh.2023.1214766

**Published:** 2023-09-15

**Authors:** Ralitsa Raycheva, Kostadin Kostadinov, Elena Mitova, Nataliya Bogoeva, Georgi Iskrov, Georgi Stefanov, Rumen Stefanov

**Affiliations:** ^1^Department of Social Medicine and Public Health, Faculty of Public Health, Medical University of Plovdiv, Plovdiv, Bulgaria; ^2^Bulgarian Association for Promotion of Education and Science, Institute for Rare Disease, Plovdiv, Bulgaria

**Keywords:** rare disease registry, European Reference Networks (ERNs), electronic health records, issues, limitations, machine learning, artificial intelligence

## Abstract

**Background:**

Given the increased availability of data sources such as hospital information systems, electronic health records, and health-related registries, a novel approach is required to develop artificial intelligence-based decision support that can assist clinicians in their diagnostic decision-making and shorten rare disease patients’ diagnostic odyssey. The aim is to identify key challenges in the process of mapping European rare disease databases, relevant to ML-based screening technologies in terms of organizational, FAIR and legal principles.

**Methods:**

A scoping review was conducted based on the PRISMA-ScR checklist. The primary article search was conducted in three electronic databases (MEDLINE/Pubmed, Scopus, and Web of Science) and a secondary search was performed in Google scholar and on the organizations’ websites. Each step of this review was carried out independently by two researchers. A charting form for relevant study analysis was developed and used to categorize data and identify data items in three domains – organizational, FAIR and legal.

**Results:**

At the end of the screening process, 73 studies were eligible for review based on inclusion and exclusion criteria with more than 60% (*n* = 46) of the research published in the last 5 years and originated only from EU/EEA countries. Over the ten-year period (2013–2022), there is a clear cycling trend in the publications, with a peak of challenges reporting every four years. Within this trend, the following dynamic was identified: except for 2016, organizational challenges dominated the articles published up to 2018; legal challenges were the most frequently discussed topic from 2018 to 2022. The following distribution of the data items by domains was observed – (1) organizational (*n* = 36): data accessibility and sharing (20.2%); long-term sustainability (18.2%); governance, planning and design (17.2%); lack of harmonization and standardization (17.2%); quality of data collection (16.2%); and privacy risks and small sample size (11.1%); (2) FAIR (*n* = 15): findable (17.9%); accessible sustainability (25.0%); interoperable (39.3%); and reusable (17.9%); and (3) legal (*n* = 33): data protection by all means (34.4%); data management and ownership (22.9%); research under GDPR and member state law (20.8%); trust and transparency (13.5%); and digitalization of health (8.3%). We observed a specific pattern repeated in all domains during the process of data charting and data item identification – in addition to the outlined challenges, good practices, guidelines, and recommendations were also discussed. The proportion of publications addressing only good practices, guidelines, and recommendations for overcoming challenges when mapping RD databases in at least one domain was calculated to be 47.9% (*n* = 35).

**Conclusion:**

Despite the opportunities provided by innovation – automation, electronic health records, hospital-based information systems, biobanks, rare disease registries and European Reference Networks – the results of the current scoping review demonstrate a diversity of the challenges that must still be addressed, with immediate actions on ensuring better governance of rare disease registries, implementing FAIR principles, and enhancing the EU legal framework.

## 1. Introduction

### 1.1. Rationale

A rare disease (RD) is a health condition that affects a small number of people compared with other prevalent diseases in the general population (1). A disease is deemed rare in the European Union (EU) if it affects no more than 5 people out of every 10,000 (2). Although rare diseases individually afflict a small number of people, collectively they may affect over 6% of the world’s population (3). Worldwide, more than 400 million people have RDs, according to the World Health Organization (WHO) (4).

Around 80% of RDs are of genetic origin and predominantly affect children, with 70% having exclusively pediatric onset, which emphasizes the importance of genetic screening for timely RDs diagnosis. Recent advances in genomic sequencing technologies and molecular gene therapies have enhanced diagnosis and expanded treatments (5).

Many RDs are severe, chronic, and life-threatening and there are no approved therapies for over 90% of these disorders (6). Therefore, in recent years, there is increased recognition of RDs as a global public health problem with high medical, psychological, and social impacts as well as an excessive economic burden to patients, families, and healthcare systems (7).

Finding the proper diagnosis presents a significant barrier in the treatment of RDs. Patients with RDs report many years of convoluted journey with multiple misdiagnoses and an average diagnosis delay of up to 8 years (8). A non-specific clinical presentation, involving multiple organ systems that appear unrelated, a general lack of awareness and physician training regarding RDs, the absence of standard diagnostic criteria, the scarcity of specialists, and the disorganized patient journeys through the healthcare systems are just a few of the factors that contribute to the diagnostic odyssey that many patients with RDs experience. All these elements result in information loss, raise the risk of errors, and occasionally limit access to diagnostic tools (9).

Initiatives and networks that aim to pool data and knowledge about rare diseases so that healthcare providers can quickly access and communicate pertinent information are viable strategies for enhancing medical care for people with rare diseases (10). Orphanet (11), which offers information on disease epidemiology, linked genes, inheritance types, disease onsets, or references to terminologies, as well as links to specialist centers, patient organizations, and other resources, is one of the most comprehensive knowledge bases for rare diseases. Other European initiatives include the European Reference Networks (ERNs), which offer an IT infrastructure that enables healthcare professionals to collaborate on virtual panels to exchange knowledge and choose the best treatments (12), the European Joint Programme on Rare Diseases (EJP RD), a multinational initiative, and RDConnect, which combines registries, biobanks, genetic data, and bioinformatics tools to provide a central resource for research on rare diseases (12).

Advances in information technology, particularly in the areas of artificial intelligence (AI) and machine learning, are significant factors that can improve the situation for patients with rare diseases in addition to these joint initiatives and international platforms. AI and machine learning are being used more and more in healthcare and medicine (13, 14). For example, there are online tools for the diagnosis of genetic or rare diseases, using phenotype concept, such as Phenomizer,^1^ or RDAD (Rare Disease Auxiliary Diagnosis system)^2^ aimed to build diagnostic models using phenotypic similarity and machine learning. Another example is the RD-Connect Genome-Phenome Analysis Platform,^3^ an online tool for diagnosis and gene discovery in rare disease research. In order to create decision support systems that could aid clinicians in making diagnostic decisions, particularly in RDs, it is necessary to expand the availability of data sources, such as hospital information systems (HISs), electronic health records (EHRs), and health-related registries. A review of clinical decision support tools using artificial intelligence (AI), confirmed the importance of advanced analysis methods such as machine learning (ML) in clinical decision-making (15). For the purpose of helping diagnose people with RDs by such analysis methods, the usage of data sources based in EU countries is closely related to legal and ethical standards within the European legislative framework; it also needs to be facilitated through FAIR principles for data management (Findability, Accessibility, Interoperability, and Reusability) (16, 17). Mapping and overviewing these data sources are a step towards developing AI and ML-based tools for faster and more precise diagnostic processes in the RDs area. A definite need for evaluating European RD data sources in terms of fulfillment of FAIR principles and meeting EU regulation challenges was established, while considering the potential of RD databases in the process of genetic newborn screening and artificial intelligence (AI)-based tools, which could significantly shorten the time required for RD diagnosis (S4C project). The S4C project is focusing on finding routes for early detection of RDs via advanced information technology and clinical decision support tools, using artificial intelligence (AI) and ML, including the development of a federated metadata repository amendable to federated ML algorithms (S4C project).

### 1.2. Objectives

The aim of our scoping review is to identify key challenges in the process of mapping European rare disease databases, relevant to ML-based screening technologies in terms of organizational, FAIR and legal principles.

## 2. Methods

### 2.1. Study design

This scoping review’s reporting adheres to PRISMA-ScR [Preferred Reporting Items for Systematic Reviews and Meta-Analyses extension for Scoping Reviews (18)] (Supplementary material 1) and the JBI Manual for Evidence Synthesis (19) in accordance with the framework outlined below: (1) defining and aligning the objective/s and question/s; (2) developing and aligning the inclusion criteria with the objective/s and question/s; (3) describing the planned approach to evidence searching, selection, data extraction, and presentation of the evidence; (4) searching for the evidence; (5) selecting the evidence; (6) extracting the evidence; (7) analysis of the evidence; (8) presentation of the results; and (9) summarizing the evidence in relation to the purpose of the review, making conclusions and noting any implications of the findings. For this study, no review protocol was registered.

### 2.2. Research question

To develop a clear and meaningful research question, the Population, Concept, Context (PCC) mnemonic strategy was used as a guide (19). In our review P (population) denotes rare disease patients, C (concept) denotes organizational, FAIR and legal challenges, and C (context) denotes European rare disease databases, which are relevant for ML-based screening technologies. The primary question posed in the scoping review was: What are the key organizational, FAIR and legal challenges that have been identified in the process of European rare disease databases mapping that may impede the implementation of ML-based screening technologies for rare disease patients?

### 2.3. Sources of information and eligibility criteria

Systematic searches in indexed literature databases were conducted to identify peer-reviewed studies relevant to the scoping review’s research question. Articles were considered in three categories: (1) medical and health-related publications; (2) computer science and artificial intelligence journal publications with applications in rare disease databases; and (3) law journal publications discussing the EU health data regulatory framework. We restricted our search to a 10-year timeframe, from January 1, 2013, to November 30, 2022, because earlier publications might be irrelevant to our review. Only human-related articles disseminated in English were included. The primary study search was performed in Medical Literature Analysis and Retrieval System Online (MEDLINE) via PubMed, Scopus and Web of Science (Core Collection). Google Scholar was the database in use for secondary search, and citation searching was also performed. Grey literature publications were not included.

### 2.4. Search term definition procedure and search strategy

Three separate bibliographic searches were conducted in each of the selected databases to find relevant evidence for the research question. One search was conducted to identify evidence on the organization of rare disease databases, FAIR and legal challenges. Next, two separate searches were conducted for FAIR and legal categories: one search for FAIR (i.e., Findability, Accessibility, Interoperability, and Reuse), and one combined search for legal issues. Searches were developed based on separate search terms for each category of interest, relevant study design filters and time limitations. Because of the categories’ comprehensiveness, depth, and heterogeneity, the terms were chosen using a PCC strategy to locate the greatest number of relevant articles while also being precise enough to lower the number of false positives. The Boolean operators AND and OR, the MeSH (Medical Subject Headings), DeCS (Health Science Descriptors), and Emtree thesaurus were used to determine whether these terms were controlled, or uncontrolled descriptors indexed in the selected databases (Supplementary material 2).

### 2.5. Inclusion/exclusion criteria and study selection

Results of the searches were uploaded into Rayyan (20), a web-based application that facilitates collaboration among reviewers during the study selection process. First, software functions were used to remove duplicates of publications, then each publication was screened against the scoping review predefined inclusion and exclusion criteria (Table 1). Specifically, a publication was screened at level 1 – title and abstract review and, if it passed this stage, went on to level 2 – a full-text review. At both levels, each screened publication was reviewed by two independent researchers who had been trained in the objectives of the review. The researchers recorded their screening decisions on Rayyan website review form that was generated for each search. The third senior researcher was the only one qualified to check separately both reviewers’ selections and was able to settle the disagreements about screening decisions.

**Table 1:** Inclusion and exclusion criteria for level one (title and abstract) and level two (full-text) screenings.

Screening level	Inclusion criteria	Exclusion criteria
Title and abstract	Types of publications such as primary research, literature reviews, study protocols, commentaries, and editorials	Types of publications such as dissertations and thesis
Title and abstract	The publication has an abstract available	The publication has no abstract available
Title and abstract	The publication is written in English	The publication is written in a language other than English
Title and abstract	Articles published in the last 10 years	Articles not published in the last 10 years
Title and abstract		Duplicates
Title and abstract & Full-text	Articles containing data from countries in Europe (EU/EEA)	Articles containing data from countries different from Europe (EU/EEA)
Title and abstract & Full-text	Publications containing information about **rare disease databases:** including electronic health records (EHR), electronic medical records (EMR), hospital information systems (HISs) and registries	Publications not including information on rare disease databases
Full-text	Publications containing information about **challenges met in**: **diagnostic** process of rare diseases; RD databases’**usability** in terms of fulfilling **FAIR** data principles; RD databases meeting EU **regulation** requirements (data ownership and data sharing); RD databases answering **legal** concerns (GDPR, consents)	Publications not including information on challenges (FAIR, legal or other) in the implementation of RD databases to improve the RD patients’ diagnostic process
Full-text	Publications containing information about the **potential** of RD databases to be implemented in the process of genetic newborn **screening** and artificial intelligence (**AI**)/machine learning (**ML**)-based tools	Publications not containing information about the implementation potential of RD databases for genetic newborn screening and artificial intelligence (AI)/machine learning (ML)-based tools
Full-text		The publication is not human subject research

### 2.6. Data charting process

The data charting form is the result of all authors’ collaboration and agreement on which data items to be extracted as a guideline through the data charting process (Table 2). Basically, the data items selected reflected the research question and because of its heterogeneity, some of them were defined as subitems and logically were organized under a primary group. The data charting form included three main categories: (1) authors, year of publication, article title, objective or research question or hypotheses; (2) challenges met when mapping RD databases – organizational, FAIR and legal-related (the legal domain included legislative, regulatory and ethical issues); and (3) good practices, guidelines, recommendations to follow when mapping RD databases – general, FAIR, legal and ethical-related. Our rationale for allocating the data items was based on the topic of the articles identified in the literature search. When we read the full-text articles, we assigned them to one of the specific domains. Following that we extracted the data items, based in the specific context they were explained in – the data items reflect the domain topic included in the article. Naturally, there were overlaps between the domain data items, but the allocation was made based on the context of use in the article.

**Table 2:** Selected data items for data charting.

DOMAINS	Data item	Subitems
**Organizational**		
	Quality of data collection	
	Long term sustainability	Private vs. public funding
	Governance, planning and design	
	Lack of harmonization and standardization	Data heterogeneity and siloed research
	Privacy risks and small sample size	
	Data accessibility and sharing	
	Good practices, guidelines, and recommendations	
**FAIR**		
	Findable	
	Accessible	
	Interoperable	
	Reusable	
	Good practices, guidelines, and recommendations	
**Legal**		
	Digitalization of health	
	Research under GDPR and member state law	Cross-border transfers of personal data
	Data protection by all means	Data subject rights and consent
		Genetic data and genomic data
		Primary and secondary (re-) use of data
		Pseudonymous and anonymous data
	Data management and ownership	
	Trust and transparency	
	Good practices, guidelines, and recommendations	

### 2.7. Synthesis of results

The data charting results were organized and assessed to provide an overview of the procedures used, the outcomes obtained, and to address the research question.

## 3. Results

### 3.1. Selection of sources of evidence

**Figure 1:**
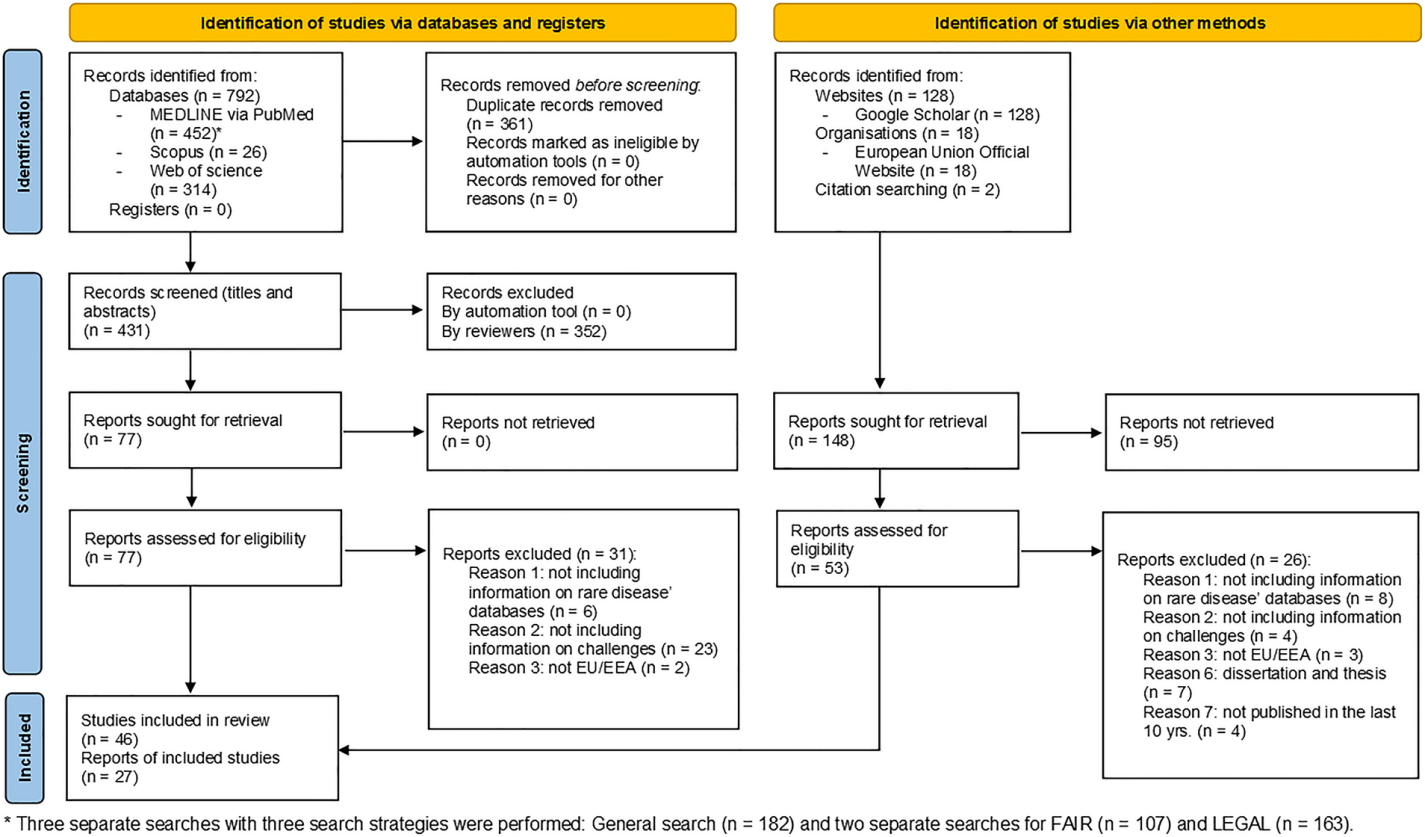
Prisma diagram of the screening process.

Three separate searches in MEDLINE via PubMed were conducted: one general (*n* = 182) and two specifics to FAIR (*n* = 102) and legal challenges (*n* = 163), respectively. The primary search yielded 792 studies in the three databases, including the identified publications in Scopus (*n* = 26) and Web of Science (*n* = 314). After merging the databases, Rayyan Software removed the duplicate records (*n* = 361), resulting in 431 articles available for a title and abstract screening. At this phase, 352 publications were excluded mainly due to: (1) lack of information relevant to the research question; (2) not EU/EEA; (3) incorrect publication type; and (4) lack of full-text availability, etc. The remaining 77 articles’ texts were then read in full. Finally, 46 studies were included and analyzed because they all matched the inclusion criteria. The additional search identified 128 articles in Google Scholar, 18 legal documents from the EU Official website and two citations. After duplicate removal 53 articles were considered eligible for full-text screening, resulting in 27 reports being included. As a result, 73 articles were retained at the end of the entire process. Both primary and secondary search article flows were illustrated on the PRISMA diagram (Figure 1) for the three phases of the process: identification, screening, and inclusion (21).

### 3.2. Characteristics of sources of evidence and results of individual sources of evidence

The complete list of all 73 articles with their metadata elements extracted is available in Supplementary material 3.

### 3.3. Synthesis of results

**Figure 2:**
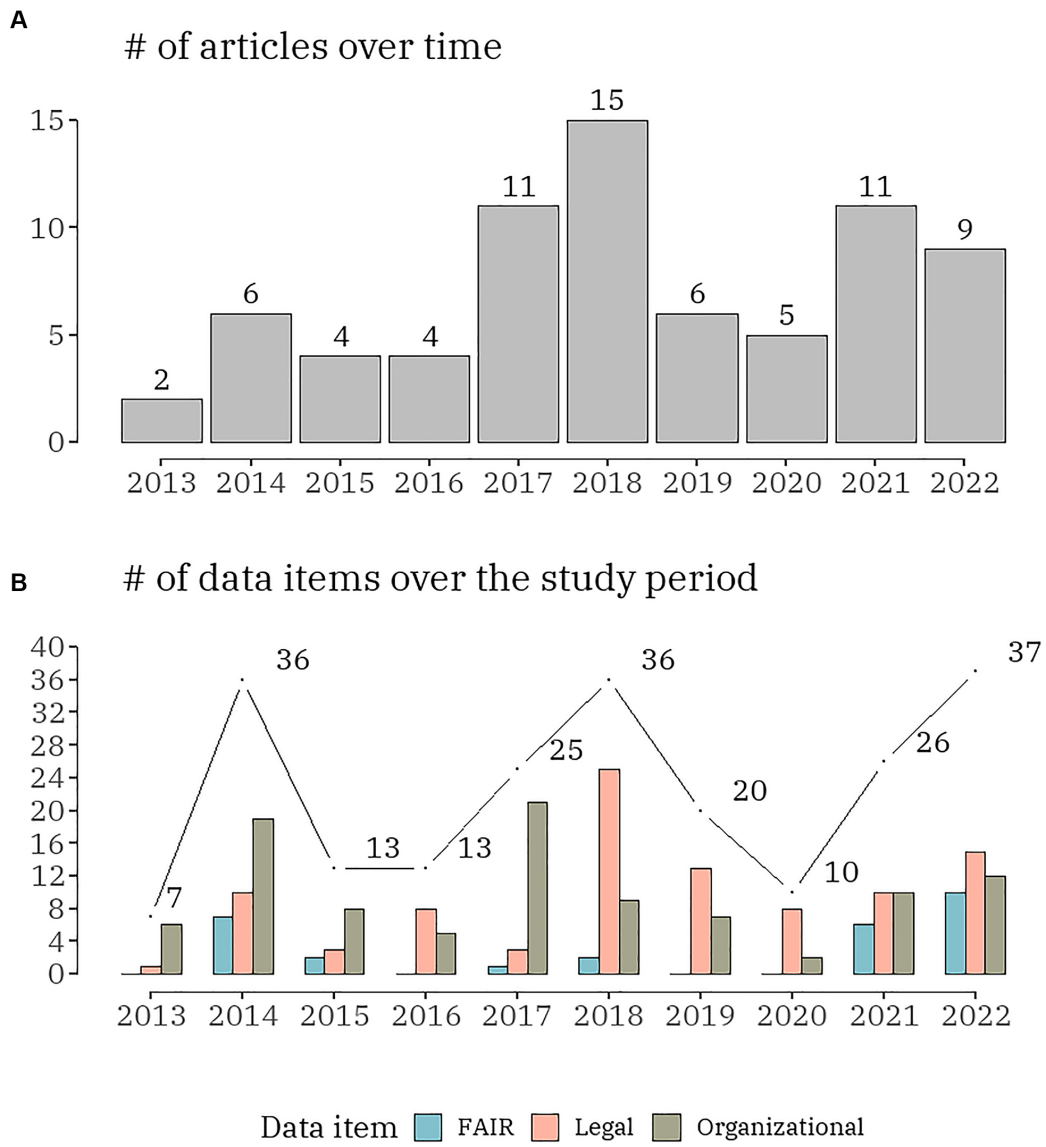
The number of publications **(A)** and the number of data items **(B)**, distributed by the three domains (organizational, FAIR and legal) per year.

Among the 73 articles published between 2013 and 2022, more than 60% (*n* = 46) were published in the last 5 years and all originated from European countries. The number of annual publications in organizational, FAIR and legal challenges in the context of rare disease databases were charted in Figure 2. Beginning with 2 (2.7%) in 2013 and increasing to 4 (5.5%) in 2015 and 2016, the number of publications reporting any challenges was rather modest. There was a significant increase in 2017, with 11 (15.1%) articles, followed by the highest number of publications (20.5%, *n* = 15) in 2018. The following two years demonstrated a decline: 2019–6 (8.2%) and 2020–5 (6.8%). There has been an increase in publications over the past two years: 11 (15.1%) in 2021 and 9 (12.3%) in 2022. The number of yearly distributions of the data items by organizational, FAIR and legal challenges in the context of rare disease databases was visualized in Figure 2. Over the ten-year period of the scoping review, there was a clear cycling trend in the publications, with a peak of challenge reporting every four years. Within this trend, the following dynamic was identified: except for 2016, organizational challenges dominated the articles published up to 2018; legal challenges were the most discussed topic from 2018 to 2022.

**Figure 3:**
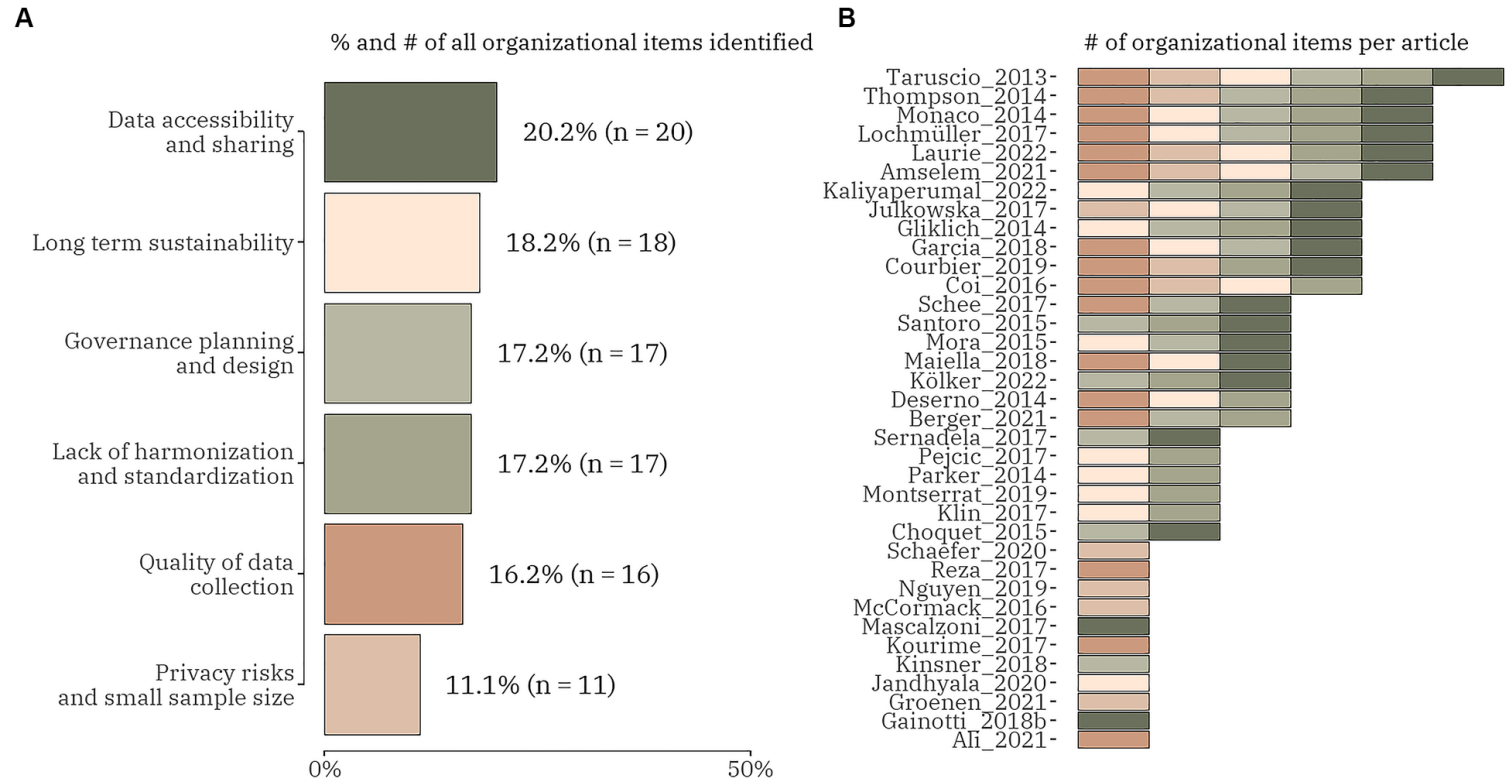
The proportion (number) of organizational domain data items **(A)** and the distribution of the items within each identified publication (*n* = 36) **(B)**.

Regarding the organizational challenges, six different categories were focused on, i.e., (1) data accessibility and sharing; (2) long-term sustainability; (3) governance, planning and design; (4) lack of harmonization and standardization; (5) quality of data collection; and (6) privacy risks and small sample size (Figure 3). From the total of 73 publications 36 (49.3%) included any of the organizational data items. The data items were relatively evenly distributed with the smallest proportion observed for the last one of the listed – 11.1% (*n* = 11). Only one article discussed all organizational challenges (22). Five articles combined five of the data items all including the quality of data collection and data accessibility and sharing (12, 23–26). Six articles analysed four organizational data items mainly focused on data accessibility and sharing and lack of harmonization and standardization (27–32). Seven studies debate about three of the data items (33–39). Six publications confer about two organizational challenges: in four articles these were – long-term sustainability and governance, planning and design (40–43) and in the other two – lack of harmonization and standardization and data accessibility and sharing (44, 45). All other 11 articles were exchanging views on one data item only (10, 46–55). Funding as a subitem of long-term sustainability was discussed in 6 studies (25, 26, 29, 31, 42, 56). Data heterogeneity and siloed research were examined as a subitem of a lack of harmonization and standardization in all articles in which the main item was included.

**Figure 4:**
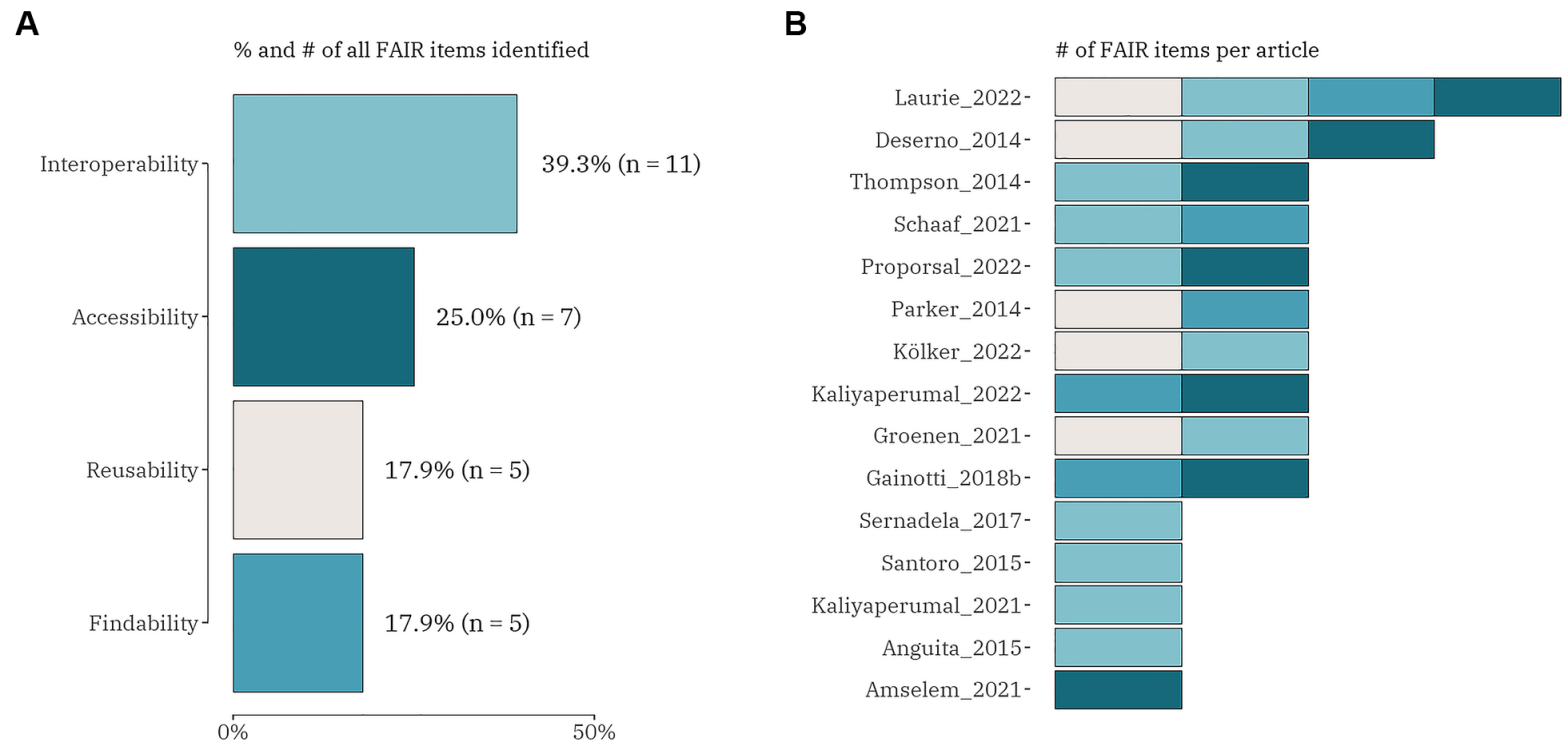
The proportion (number) of FAIR domain data items **(A)** and the distribution of the items within each identified publication (*n* = 15) **(B)**.

Regarding the FAIR challenges, four different categories were focused on, i.e., (1) findable; (2) accessible sustainability; (3) interoperable; and (4) reusable (Figure 4). From the total of 73 publications 15 (20.5%) included any of the FAIR data items. Only one article included all four data items (24) and only one discussed three FAIR challenges, but not including the findable item (34). Eight articles combined two out of four FAIR data items in different combinations: five studies concentrated on interoperability with reusability (35, 47); with accessibility (12, 57) and with findability (58); three articles focused on findable – with accessible (32, 47) and with reusability (42). All five other publications explored only one FAIR data item, with interoperability (38, 45, 59, 60) outweighing accessibility (23).

**Figure 5:**
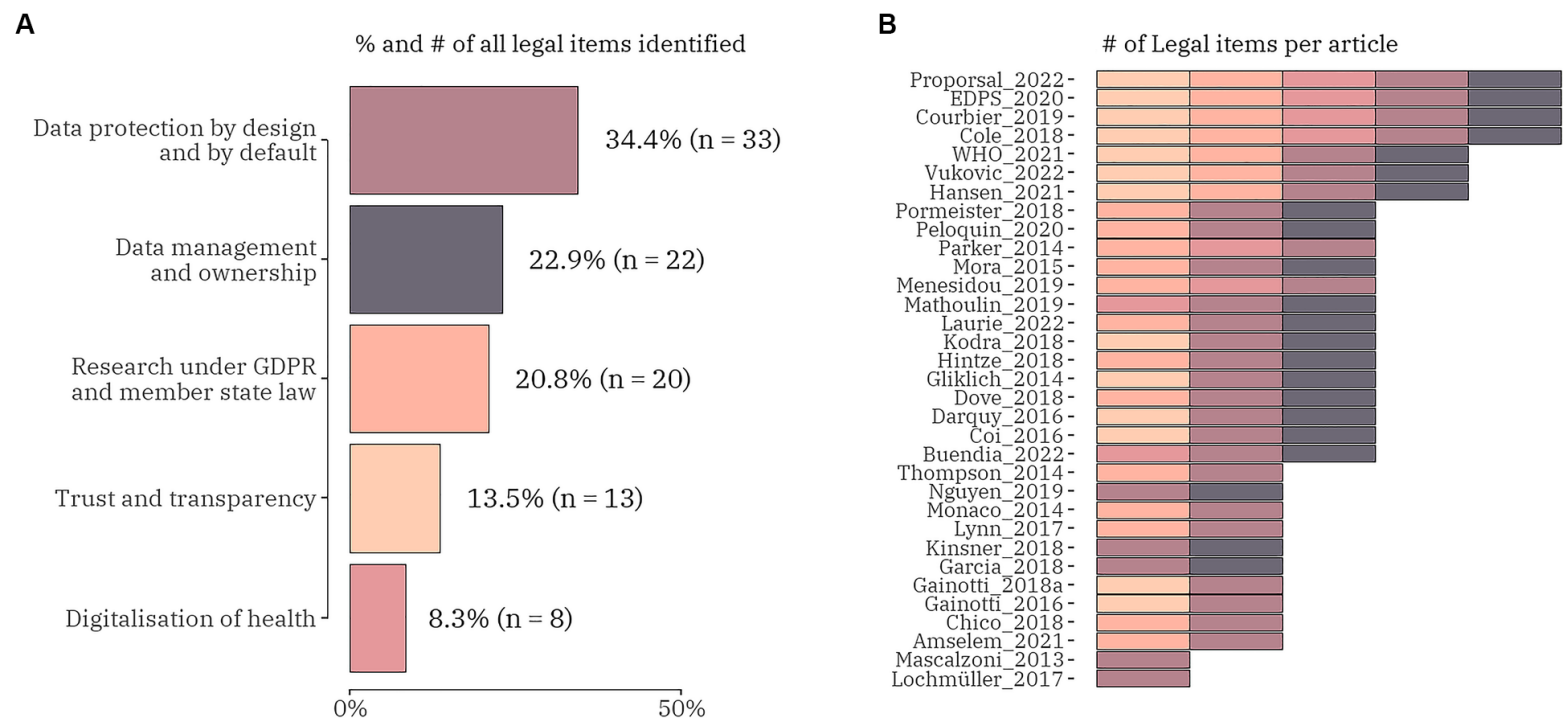
The proportion (number) of legal domain data items **(A)** and the distribution of the items within each identified publication (*n* = 33) **(B)**.

Regarding the legal challenges, five different categories were focused on, i.e., (1) data protection by all means; (2) data management and ownership; (3) research under GDPR and member state law; (4) trust and transparency; and (5) digitalization of health (Figure 5). From the total of 73 publications 33 (45.2%) included any of the legal data items. Four articles included all five legal data items (28, 57, 61, 62). There were three articles discussing the same combination of four legal challenges: digitalization of health, research under GDPR and member state law, data protection by all means, and data management and ownership (63–65). Most of the studies (*n* = 14) were focused on three of the legal data items: 6 publications blended research under GDPR and member state law, data protection by all means and data management and ownership (24, 37, 66–69); 4 publications mixed data protection by all means, data management and ownership and trust and transparency (27, 30, 56, 70); and 2 sets of the following combinations – digitalization of health, data protection by all means and data management and ownership (71, 72); digitalization of health, research under GDPR and member state law and data protection by all means (17, 42). Of the remaining 12 publications two were entirely focused on data protection by all means (26, 73) and the rest mixed two legal data items in the following patterns: research under GDPR and member state law and data protection by all means (12, 23, 25, 74, 75); data protection by all means and data management and ownership (29, 50, 54); and data protection by all means and trust and transparency (76, 77). The data item with the highest proportion (45.0%, *n* = 33) included in all articles under review was data protection by all means. It contained four subitems: data subject rights and consent (81.8%, *n* = 27); genetic data and genomic data (33.3%, *n* = 11); primary and secondary (re-)use of data (39.4%, *n* = 13); and pseudonymous and anonymous data (60.6%, *n* = 20). Cross-border transfer of personal data was added as a subitem to research under GDPR and member state law data item and was highlighted as a challenge in 11 studies.

**Figure 6:**
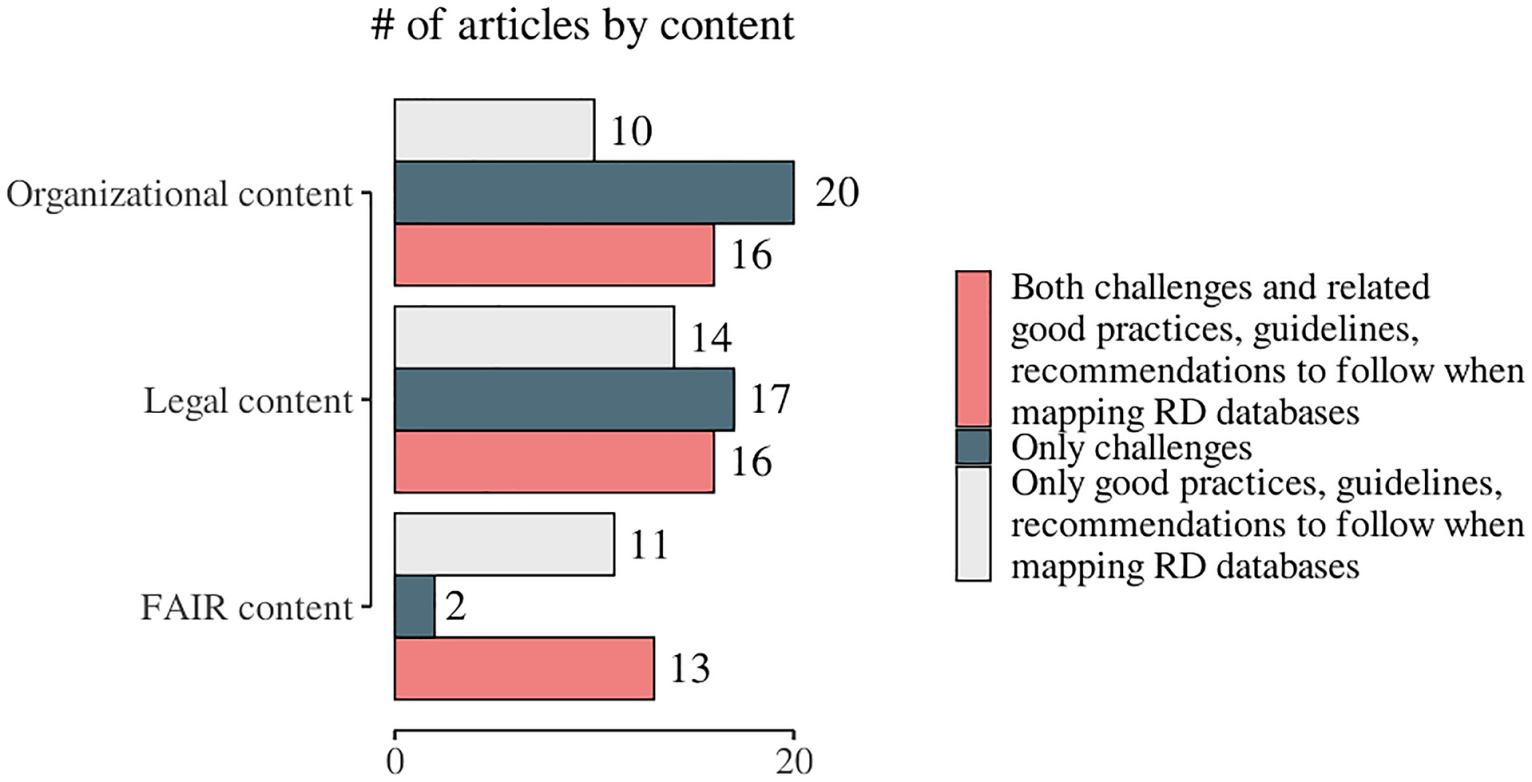
Distribution of the identified challenges and/or good practices, guidelines, and recommendations by the domains of the study.

We observed a specific pattern repeated in all domains during the process of data charting and data item identification – in addition to the outlined challenges, good practices, guidelines, and recommendations were also discussed. The proportion of publications addressing only good practices, guidelines, and recommendations for overcoming challenges when mapping RD databases in at least one domain was calculated to be 47.9% (*n* = 35). The articles that highlighted good practices, guidelines, and recommendations for overcoming any of the one domain’s challenges but did not provide solutions to any of the other two domains’ issues under consideration were 25 out of 35 (71.4%). Only three studies provided suggestions on good practices, guidelines, and recommendations for all three domains (78–80). Only challenges were discussed in 53.4% (*n* = 39) of the articles included in the present scoping review. We identified both challenges and related good practices, guidelines, and recommendations in 61.6% (*n* = 45). The distribution of the challenges and/or good practices, guidelines, and recommendations by the domains’ content is presented in Figure 6 and publication-based detailed information is included in Supplementary material 3. We identified 11 (15.0%) papers that were broadly focused on good practices, guidelines, and recommendations but did not cover any of the specific data items chosen for this scoping review (Supplementary material 3; list numbers 63–73).

## 4. Discussion

### 4.1. Summary of evidence

With the evolution of science and new technologies, both health professionals and patients expect researchers to share data in order to speed up the pathway to a diagnosis and, ultimately, effective treatments. The key organizational challenges that have been identified in the process of mapping European rare disease databases, reflect the variety of issues faced during their development, such as data quality, sustainability, funding, and governance. Quality of data collection, including the need for quality control and recommendations, is discussed in over 16% of assessed articles. Data collection on patients’ diseases is challenging as there are few patients for each disease, which are spread over wide geographic regions – biological collections and databases are typically local, limited, fragmentary, and not always subject to quality control (23). While many articles (>17%), comment on the lack of harmonization and standardization, including siloed research and data heterogeneity, a major challenge remains data accessibility and sharing, mentioned in over 20% of the assessed publications. In rare diseases research it is essential to share information internationally, as there is a need to find similar cases in this field with scarce patient numbers. On the other hand, the privacy risks and protection of data, ownership, and control, commented in >11% of articles, deserve consideration when looking for best practices and solutions. Other barriers to sharing RD data include the cost of making and maintaining the data interoperable, discoverable, and accessible (24). RD registries are limited by funding and resources and budgets are often exhausted by data collection and processing tasks alone (56). Regarding future challenges, multiple authors discuss long-term sustainability. In the case of ERNs, a need for their integration into the healthcare systems of the countries is established (41). The long-term sustainability of RD research is linked to funding and investment, therefore a higher rate of scientific publications and evidence generation in relation to private funding is commented on by Jandhyala et al. (49). The implementation of ML-based screening technologies for people with rare diseases may be hampered by the need to optimize the use of RD databases for research, which demands a significant amount of work and is further complicated by regulatory restrictions.

The FAIR principles (Findable, Accessible, Interoperable, and Reusable) provide a framework for ensuring that data are effectively managed and shared in a way that maximizes their utility. In the context of rare diseases, where data can be particularly sparse, ensuring that databases adhere to FAIR principles is crucial to facilitate research and accelerate progress in the field (12, 48, 60). According to the scope of the review, adherence to FAIR principles as a topic is more commonly used in recently published articles (2021–2022). The “FAIR-ness” of the database included in the articles varies widely. Some databases, such as the FAIR registry for vascular anomalies (VASCA centres), have made significant efforts to ensure that their data are FAIR in all their dimensions (47). Several articles provide in-depth concepts of the technical and methodological requirements towards “FAIR-ification” of the rare diseases’ registries at the European level (33, 47, 56, 60, 80). Most of the founded articles address the interoperability achieved mainly by APIs development as a tool allowing data to be easily accessed and integrated with other systems (12, 24, 45). The less frequently found data item is findability. However, articles identified with this data item share a common recommendation emphasizing the use of standardized terminology to enhance the findability of its data (24, 42, 47, 58, 60). Despite these recommendations, there are still challenges to achieve full FAIR compliance for rare disease databases. Data can be sparse, and there is often a lack of standardization in terminology and metadata (23). In addition, smaller or less well-funded databases may lack the resources needed to fully implement the FAIR principles, mostly because of improper database design (12), lack of security access technical solutions (23, 34, 57) or unachievable interoperability (48, 57). To overcome these challenges, stakeholders in the rare disease community should collaborate to support the development and maintenance of FAIR-compliant databases. Such collaboration is proven to be efficient in delivering open-source FAIR technical solutions for small databases (24, 34, 60). Further, initiatives should involve investing in standardization efforts, such as the use of common data elements and ontologies which provide a technique to explain concepts using vocabulary that is arranged in a hierarchical or tree structure (59), as well as providing funding and other resources to support the development of new databases and the access improvement of existing ones (45). In addition, efforts to promote data sharing and collaboration, such as the use of common data repositories or tools such as dynamic data management planning questionnaires, could help to enhance the interoperability of rare disease data and support progress in the field (47, 59, 81).

Development of information systems with a variety of data architectures and innovative fields such as Big Data, Machine Learning, and Artificial Intelligence provides a wealth of opportunities for more efficient collection, use, and sharing of health data, but also poses new challenges for privacy and data security (28, 71, 72). It is therefore not surprising that 2018, the year the General Data Protection Regulation (the GDPR or the Regulation) became effective, had the greatest number of articles addressing legal challenges (61, 66, 67, 74). Legal issues undoubtedly continue to appear in publications, though not to the same extent, until the end of the study period. Despite being perceived as a modern, fit-for-purpose Regulation that will ensure a consistent and high level of protection for European citizens and remove barriers to personal data flows within the EU, 20 publications identified the GDPR and the sub-category of cross-border transfers of personal data as a challenge to clinical practice and scientific research. Understanding the ‘purpose’ of data collection and its subsequent use is critical in understanding the legal requirements of how those data are managed and protected (61), which explains a large number of articles (*n* = 22) bringing up data management and ownership constraints (57, 62, 63, 65, 68). Legal compliance is unquestionably at the top of every rare disease registry list of reasons for implementing information security measures to safeguard sensitive data. This is the reason why most of the publications (34.3%, *n* = 33) highlighted data protection as a paramount challenge. Moreover, the authors addressed different subcategories. There is a certain level of uncertainty and disagreement as to whether genomic data are also covered by the definition of genetic data in EU legislation (63, 69, 75). The principle of purpose limitation is one of the core principles of data protection, as data controllers must specify the exact purpose before beginning processing activities. The purpose limitation principle is not absolute in the case of health data, as a secondary use of health data is critical for the management and improvement of public health systems (67, 68, 75). Anonymization and pseudonymization are safeguarding tools that ensure data sharing safety, but only if their implementation is deliberately designed – that is, the basic requirements (context) and goal(s) of the anonymization procedure must be clearly set out to accomplish the targeted anonymization while generating some meaningful data (28, 57, 62, 67, 68, 77). The most central of all challenges that are in the scope of data sharing and protection is consent. Data subjects’ rights have been the focus of issues discussed throughout the overall period of the study (27, 28, 30, 61, 64, 71, 73, 74, 76). The overarching assumption is that patients are willing to contribute their data but are concerned about data sharing (28, 30, 70) and the risk of identifiable data is increased in the context of rare diseases (54). The need for improving informed consent processes in international collaborative rare disease research is broadly discussed, namely, there is a need for effective consent in order to conduct effective research. To achieve this aim, the procedure shall address possible ethical and legal hurdles that could hamper research in the future, including opt-in, re-consent and opt-out strategies (17, 54, 76). Trust is a key issue for patients involved in rare disease research, and it could be argued that this becomes even more apparent in data sharing, with the onus on researchers, institutions, and collaborations to recognize this as a responsibility (57, 64). There is another aspect that should be considered – although patients are the actual owners of their health data, there might be factors that prevent timely data sharing, apart from patients’ consent. Challenges could arise from the reluctancy of clinicians to share research data because of publication pressure, intellectual property, and competition (82).

The European Union has been addressing the digitalization of health as building trust between the Member States by establishing laws, regulations, directives, and other acts for data protection/security, usage, processing and sharing. Although proactive policymaking, there are challenges that should be overcome. In its European strategy for data the European Commission outlines the future steps to overcome “the fragmented landscape of digital health services, especially when provided cross-border to exchange health data; link and use, through secure, federated repositories, specific kinds of health information, such as EHRs, genomic and digital health images, in compliance with the GDPR” (83). The European Data Strategy, which aims to establish a single market for data by enabling simpler and more secure access and usage of data, was introduced by the European Commission in order to safeguard Europe’s competitiveness and data sovereignty. One of the objectives of the Commission for 2019–2025 is the creation of a multisectoral European Health Data Space (EHDS), with the health sector being one of those involved. The EHDS expands the main use of health data, regulates the secondary use of health data, and adds rules for reusing health data, all of which are based on the framework set forth by the Data Governance Act (83).

In October 2021, a new international Innovative Medicines Initiative (IMI) project, Screen4Care, was formally launched with a focus on accelerating diagnosis for Rare Disease in EU patients based on two central pillars: genetic newborn screening and digital technologies. The challenges identified in this study will be utilized to develop a questionnaire that would collect specific details about the technical, legal, and business aspects of the data that rare disease organizations work with. Thus, the collected information will serve the Screen4care goal of significantly shortening the rare disease patients diagnosis odyssey by implementing advanced analysis methods such as machine learning and Artificial Intelligence (84).

### 4.2. Limitations

We should outline some limitations identified throughout the scoping review process. First, the time frame selected (from January 2013 to November 2022) and the language restriction to only papers written in English may have influenced the final sample of articles. Second, the grey literature was not considered and using only PubMed, Scopus and Web of Science as data sources but not covering unpublished literature may have limited the search’s scope. However, records identified via websites, organizations and citation searches helped us in minimizing this limitation. Furthermore, the data charting process encompassed a broad and heterogeneous list of items, which were organized into main groups and subgroups under the three domains that addressed the research question. Thus, some of the data items’ importance might have been underestimated or overestimated. A more detailed assessment of best practices and solutions to identified challenges could be of interest to a future study. The S4C project scope is defined by EU Commission funding grant requirements and is thus limited to European rare disease databases. However, we do not anticipate substantial differences in results from non-European databases.

## 5. Conclusion

Digital transformation in healthcare altered the interaction between health professionals and patients, the health data flow among providers and the decision-making process about treatments and health outcomes, especially in the field of rare diseases. It brought automation, electronic health records, hospital-based information systems, biobanks, rare disease registries, European Reference Networks, etc. Despite the opportunities provided by innovation, the results of the current scoping review demonstrate the diversity of the challenges that must still be addressed, with immediate actions on (1) ensuring better data quality, sustainability, funding, and governance of rare disease registries; (2) establishing and maintaining FAIR-compliant databases; and (3) and adapting the legal framework for trustworthy data collection, access, uses, and interoperability acceleration across Europe while developing health data infrastructures and shaping the future landscape of digital health services. Our findings, which are based on 73 publications from a 10-year timeframe and a broad research question, could serve as a good starting point for narrow-focused systematic reviews and in-depth analysis of challenges that are underrepresented in the identified studies.

## Data availability statement

The raw data supporting the conclusions of this article will be made available by the authors, without undue reservation.

## Author contributions

RS: conceptualization. RR and NB: methodology. RR, NB, and KK: software and data curation. RS, GS, and EM: validation. RR, NB, EM, and KK: formal analysis, investigation, and resources. RR, EM, and KK: writing—original draft preparation. GI, GS, and RS: writing—review and editing and supervision. KK and RR: visualization. GS: project administration and funding acquisition. All authors contributed to the article and approved the submitted version.

## Funding

The Screen4Care EU-IMI project has received funding from the Innovative Medicines Initiative 2 Joint Undertaking (JU) under grant agreement No 101034427. The JU receives support from the European Union’s Horizon 2020 research and innovation programme and EFPIA.

## Conflict of interest

The authors declare that the research was conducted in the absence of any commercial or financial relationships that could be construed as a potential conflict of interest.

## Publisher’s note

All claims expressed in this article are solely those of the authors and do not necessarily represent those of their affiliated organizations, or those of the publisher, the editors and the reviewers. Any product that may be evaluated in this article, or claim that may be made by its manufacturer, is not guaranteed or endorsed by the publisher.

## Footnotes

^1^http://compbio.charite.de/phenomizer

^2^http://www.unimd.org/RDAD/

^3^https://rd-connect.eu/what-we-do/omics/gpap/
