# Supplementary material for: Challenges in mapping European rare disease databases, relevant for ML-based screening technologies in terms of organizational, FAIR and legal principles: scoping review
Source: Front Public Health. 2023 Sep 15;11:1214766. doi: 10.3389/fpubh.2023.1214766 (PMC10540868; doi:10.3389/fpubh.2023.1214766)
Supplement: Supplementary file 2 [file Data_Sheet_2.PDF]

Supplementary Material 2

Challenges in mapping European rare disease databases, relevant for ML-based screening technologies in terms of organizational, FAIR and legal principles: Scoping review

Ralitsa Raycheva1,2, Kostadin Kostadinov1,2, Elena Mitova2, Nataliya Bogoeva2, Georgi Iskrov1,2, Georgi Stefanov2, Rumen Stefanov1,2

*** Correspondence:** Ralitsa Raycheva – raycheva@raredis.org

1 Supplementary: Search strategies

Supplementary file_2. Search strategies

DATABASE	SEARCH STRATEGY
**MEDLINE/Pubmed**	
General search	(((rare disease*[MeSH Major Topic]) AND (diagnos* or undiagnos* or challenge* or screening, newborn or ai artificial intelligence or machine learning[MeSH Terms]) AND (data or database* or FAIR or findable or accessible or interoperable or reusable or legal or GDPR or regulation* or regulatory or law* or recommendation* or sharing or protection or consent or guideline*[MeSH Terms]) AND (registry or registries or record* or hospital* or system[MeSH Terms]))) AND (Europe or European or EU) Filters: in the last 10 years, English, Humans
Narrowed Search (FAIR)	(((rare disease*[MeSH Major Topic]) AND (data or database* or record* or hospital* or registry or registries[MeSH Terms])) AND (FAIR or findable or accessible or interoperable or reusable[MeSH Terms])) AND (Europe or European or EU[MeSH Terms])
Narrowed Search (Legal)	(((rare disease*[MeSH Major Topic]) AND (data or database* or record* or hospital* or registry or registries[MeSH Terms])) AND (legal or GDPR or regulation* or regulatory or recommendation* or data sharing or consent* or guideline*[MeSH Terms])) AND (Europe or European or EU[MeSH Terms])
**Scopus**	#1: TITLE-ABS-KEY((“Rare Diseases” OR “Disease, Rare” OR “Rare Disease” OR “Orphan Diseases” OR “Disease, Orphan” OR “Orphan Disease”))<br>#2: TITLE-ABS-KEY((“Diagnosis” OR “Undiagnos” OR “Challenge” OR “Screening, newborn” OR “AI” OR “Artificial intelligence” OR “machine learning”))<br>#3: TITLE-ABS-KEY((“Data” OR “Database” OR “FAIR” OR “Findable” OR “Accessible” OR “nteroperable” OR “Reusable” OR “Legal” OR “GDPR” OR “Regulation” OR “Regulatory” OR “Law” OR “Recommendation” OR “Sharing” OR “Protection” OR “Consent” OR “Guideline”))<br>#4: TITLE-ABS-KEY((“Registry” OR “Registries” OR “Record” OR “Hospital” OR “System”))<br>#5: TITLE-ABS-KEY((“Europe” OR “EU”))<br>#6: PUBYEAR AFT 2012<br>#7: #1 AND #2 AND #3 AND #4 AND #5 AND #6
**Web of science**	(P)—Population<br>#1: (TS=(rare diseases* OR disease rare* OR rare disease* OR orphan diseases* OR disease orphan* OR orphan disease*))<br>#2: (TS=(persons* OR person* OR people* OR patient* OR patients* OR human*))<br>#3: #1 AND #2<br>(C)—Concept<br>#4: (TS=(data or database* or FAIR or findable or accessible or interoperable or reusable or legal or GDPR or regulation* or regulatory or law* or recommendation* or sharing or protection or consent or guideline))<br>(C)—Context

	#5: (TS=(diagnose* or undiagnosed* or challenge* or screening, newborn or AI or artificial intelligence or machine learning))<br>#6: (TS=(registry or registries or record* or hospital* or system))<br>#7: (TS=(Europe or European or EU))<br>#8: #5 AND #6 AND #7<br>#9: #3 AND #4 AND #8
**Google scholar**	(“Rare Diseases”) AND (“Persons”) AND (“Challenge”) AND (“Machine Learning”) AND (“FAIR” OR “Findable” OR “Accessible” OR “Interoperable” OR “Reusable” OR “Legal” OR “GDPR” OR “Regulation” OR “Regulatory” OR “Law” OR “Recommendation” OR “Sharing” OR “Protection” OR “Consent” OR “Guideline”) AND (“Registry” OR “Registries” OR “Hospital Record” or “Hospital system”) AND (“Europe” OR “European” OR “EU”)
