# Supplementary material for: Challenges in mapping European rare disease databases, relevant for ML-based screening technologies in terms of organizational, FAIR and legal principles: scoping review
Source: Front Public Health. 2023 Sep 15;11:1214766. doi: 10.3389/fpubh.2023.1214766 (PMC10540868; doi:10.3389/fpubh.2023.1214766)
Supplement: Supplementary file 3 [file Data_Sheet_3.pdf]

Supplementary Material 3

Challenges in mapping European rare disease databases, relevant for ML-based screening technologies in terms of organizational, FAIR and legal principles: Scoping review

Ralitsa Raycheva1,2, Kostadin Kostadinov1,2, Elena Mitova2, Nataliya Bogoeva2, Georgi Iskrov1,2, Georgi Stefanov2, Rumen Stefanov1,2

*** Correspondence:** Ralitsa Raycheva – raycheva@raredis.org

1 Supplementary: Metadata elements of the publications included in the SR

Supplementary file 3_Metadata elements of the publications included in the SR

N	CITATION	TITLE	OBJECTIVE	D*	C**	R***
1	Ali et al. 2021 (46)	The Quality Evaluation of Rare Disease Registries-An Assessment of the Essential Features of a Disease Registry	The aim of this international study was to survey RD registry leaders to ascertain the level of consensus amongst the RD community regarding the quality criteria that should be considered essential features of a disease registry.	FAIR	No	No
				Legal	No	No
				Organisational	Yes	Yes
2	Amselem et al. 2021 (23)	RaDiCo, the French national research program on rare disease cohorts	Depending on cohorts, the objectives are to describe the natural history of the studied RD(s), identify the underlying disease genes, establish phenotype-genotype correlations, decipher their pathophysiology, assess their societal and medico-economic impact, and/or identify patients eligible for new therapeutic approaches.	FAIR	Yes	No
				Legal	Yes	Yes
				Organisational	Yes	No
3	Anguita et al. 2015 (59)	Toward a view-oriented approach for aligning RDF-based biomedical repositories	To enhance existing RDF schema alignment techniques by providing a mechanism to properly represent elements with context-dependent semantics, thus enabling users to perform more expressive alignments, including scenarios that cannot be adequately addressed by the existing approaches.	FAIR	Yes	Yes
				Legal	No	No
				Organisational	No	No
4	Berger et al. 2021 (33)	How to design a registry for undiagnosed patients in the framework of rare disease diagnosis: suggestions on software, data set and coding system	In this paper we focus on the question on how such a registry for undiagnosed patients can be built and which information it should contain.	FAIR	No	Yes
				Legal	No	No
				Organisational	Yes	Yes
5	Buendia et al. 2022 (71)	Is it possible to implement a rare disease case-finding tool in primary care? A UK-based pilot study	This study implemented MendelScan, a primary care rare disease case-fnding tool, into a UK National Health Service population. The 2021 UK Rare Diseases Framework highlights as a key priority the need for faster diagnosis to improve clinical outcomes.	FAIR	No	No
				Legal	Yes	Yes
				Organisational	No	Yes
6	Chico et al. 2018 (74)	The impact of the General Data Protection Regulation on health research	This piece examines the impact of the Regulation on health research.	FAIR	No	No
				Legal	Yes	No
				Organisational	No	No

N	CITATION	TITLE	OBJECTIVE	D*	C**	R***
7	Choquet et al. 2015 (44)	A methodology for a minimum data set for rare diseases to support national centers of excellence for healthcare and research	The purpose of our work was to: (i) establish a consistent, interoperable national set of DEs common to all rare dis- eases; (ii) promote EHR data entry at the bedside; and (iii) facilitate the future development of EU registries by proposing an EU standard for rare disease patient based registries. To set up our F-MDS-RD, we proposed a complete methodology based on a systematic review of the literature as well as design and validation of the DEs by four different groups of experts and decision makers.	FAIR	No	No
				Legal	No	Yes
				Organisational	Yes	Yes
8	Coi et al. 2016 (27)	The Quality of Rare Disease Registries: Evaluation and Characterization	The aim of this study was to provide useful information for characterizing a quality profile for RDRs using an analytical approach applied to RDRs participating in the European Plat- form for Rare Disease Registries 2011 “2014 (EPIRARE) survey.	FAIR	No	Yes
				Legal	Yes	Yes
				Organisational	Yes	No
9	Cole et al. 2018 (61)	Legal Barriers to the Better Use of Health Data to Deliver Pharmaceutical Innovation	Not explicitly stated	FAIR	No	No
				Legal	Yes	No
				Organisational	No	No
10	Courbier et al. 2019 (28)	Share and protect our health data: an evidence based approach to rare disease patients’ perspectives on data sharing and data protection - quantitative survey and recommendations	The aim of this survey was to explore patient and family perspectives on data sharing and data protection in research and healthcare settings and develop relevant recommendations to support shaping of future data sharing initiatives in rare disease research.	FAIR	No	No
				Legal	Yes	Yes
				Organisational	Yes	No
11	Darquy et al. 2016 (70)	Patient/family views on data sharing in rare diseases: study in the European LeukoTreat project	The aim of this study was to optimize the information and consent process to meet participants expectations against the background of the LeukoTreat project database.	FAIR	No	No
				Legal	Yes	No
				Organisational	No	No
12	Deserno et al. 2014 (34)	Integrated image data and medical record management for rare disease registries. A general framework and its instantiation to the German Calciphylaxis Registry	In this paper, we address the particular needs of investigators initiating RDRs.	FAIR	Yes	Yes
				Legal	No	No
				Organisational	Yes	No
13	Dove et al. 2018 (66)	The EU General Data Protection Regulation: Implications for International Scientific Research in the Digital Era	I do so by describing and analyzing the implications of the GDPR for international scientific research that involves the processing of participants personal data.	FAIR	No	No
				Legal	Yes	Yes
				Organisational	No	No

N	CITATION	TITLE	OBJECTIVE	D*	C**	R***
14	EDPS, 2020 (62)	A Preliminary Opinion on data protection and scientific research	The document is structured as follows: first, we sketch out the landscape of scientific research in today s digital age and the issues which arise (section 2). Second, we aim to narrow down what we understand by scientific research in the GDPR (section 3). Third, we outline the wider governance framework for research in the EU within which data protection is situated, particular as regards clinical trials (sections 4 and 5). Fourth, we present a preliminary analysis of some key principles of the special regime for data processing for the purposes of scientific research as set down in the GDPR (section 6). This includes in particular the notion of consent, the presumption of compatibility and derogations to data subject rights. Finally, we point to a number of areas for further consideration (section 7).	FAIR<br>Legal<br>Organisational	No<br>Yes<br>No	No<br>No<br>No
15	European Commission 2022 (57)	Proposal for a REGULATION OF THE EUROPEAN PARLIAMENT AND OF THE COUNCIL on the making available on the Union market as well as export from the Union of certain commodities and products associated with deforestation and forest degradation and repealing Regulation (EU) No 995/2010_2021	The general objective is to ensure that natural persons in the EU have increased control in practice over their electronic health data. It also aims to ensure a legal framework consisting of trusted EU and Member State governance mechanisms and a secure processing environment.	FAIR<br>Legal<br>Organisational	Yes<br>Yes<br>No	No<br>No<br>No
16	Garcia et al. 2018 (29)	Impact of biobanks on research outcomes in rare diseases: a systematic review	We undertook a systematic review to identify and compare the impact of stand-alone registries, registries with biobanks, and rare disease biobanks on research outcomes in rare diseases.	FAIR<br>Legal<br>Organisational	No<br>Yes<br>Yes	No<br>No<br>No
17	Gainotti et al. 2016 (76)	Improving the informed consent process in international collaborative rare disease research: effective consent for effective research	To address those special concerns we tried to determine the kind of information that should be required for this type of research in international consortia in the form of core elements (CEs) required for informing patients in research.	FAIR<br>Legal<br>Organisational	No<br>Yes<br>No	No<br>Yes<br>No

N	CITATION	TITLE	OBJECTIVE	D*	C**	R***
18	Gainotti et al. 2018 (77)	Meeting Patients' Right to the Correct Diagnosis: Ongoing International Initiatives on Undiagnosed Rare Diseases and Ethical and Social Issues	In this work we suggest that, to maximize patients involvement in the search for a diagnosis and identification of new causative genes, undiagnosed patients should have the possibility to: (1) actively participate in the description of their phenotype; (2) choose the level of visibility of their profile in matchmaking databases; (3) express their preferences regarding return of new findings, in particular which level of Variant of Unknown Significance (VUS) significance should be considered relevant to them.	FAIR	No	No
				Legal	Yes	No
				Organisational	No	No
19	Gainotti et al. 2018 (47)	The RD-Connect Registry & Biobank Finder: a tool for sharing aggregated data and metadata among rare disease researchers	Here, we present the RD-Connect Registry & Biobank Finder (http://catalogue.rd-connect.eu/), a tool that helps to find RD biobanks and registries and provides information on the availability and accessibility of content in each database.	FAIR	Yes	Yes
				Legal	No	No
				Organisational	Yes	No
20	Gliklich et al. 2014 (30)	Registries for evaluating patient outcomes: a user's guide	Not explicitly stated	FAIR	No	No
				Legal	Yes	No
				Organisational	Yes	No
21	Groenen et al. 2021 (48)	The de novo FAIRification process of a registry for vascular anomalies	This article describes the complete de novo FAIRification workflow, from identifying FAIRi- fication objectives and required expertise to querying data over a FAIR Data Point.	FAIR	Yes	Yes
				Legal	No	Yes
				Organisational	Yes	Yes
22	Hansen et al. 2021 (63)	Assessment of the EU Member States' rules on health data in the light of GDPR	A study was conducted with the objective to examine and present the EU Member States rules governing the processing of health data in light of the GDPR, with the objective of highlighting possible differences and identifying elements that might affect the cross-border exchange of health data in the EU, and examining the potential for EU level action to support health data use and re-use.	FAIR	No	No
				Legal	Yes	No
				Organisational	No	No
23	Hintze et al. 2018 (67)	Comparing the benefits of pseudonymisation and anonymisation under the GDPR	In this paper, we explore the nuances introduced by the GDPR, compare the benefits of the different levels of deidentification found in the regulation, and provide practical guidance for using de-identification as a tool for addressing different GDPR compliance obligations.	FAIR	No	No
				Legal	Yes	No
				Organisational	No	No

N	CITATION	TITLE	OBJECTIVE	D*	C**	R***
24	Jandhyala et al. 2020 (49)	Factors Influencing the Generation of Evidence from Simple Data Held in International Rare Disease Patient Registries	The aim of this study was to examine selected factors and their association with evidence generation, via scientific publication, from international rare disease patient registry data	FAIR<br>Legal<br>Organisational	No<br>No<br>Yes	No<br>No<br>Yes
25	Jonker et al. 2021 (79)	Capturing Data in Rare Disease Registries to Support Regulatory Decision Making: A Survey Study Among Industry and Other Stakeholders	The objective of this study was to investigate the opinion of stakeholders about key aspects of rare disease registries that are used to support regulatory decision making and to compare the responses of employees from industry to other stakeholders.	FAIR<br>Legal<br>Organisational	No<br>No<br>No	Yes<br>Yes<br>Yes
26	Julkowska et al. 2017 (31)	The importance of international collaboration for rare diseases research: a European perspective	This paper focuses on the efforts in the RDs field in Europe with some additional insights into international activities through the perspective of the International Rare Diseases Research Consortium (IRDiRC).	FAIR<br>Legal<br>Organisational	No<br>No<br>Yes	No<br>No<br>No
27	Kaliyaperumal et al. 2021 (60)	Enabling FAIR Discovery of Rare Disease Digital Resources	In this paper, we propose a design to implement common APIs as a complement to resources that apply RDF to implement FAIR principles. The Orphanet data catalogue was used as an example.	FAIR<br>Legal<br>Organisational	Yes<br>No<br>No	Yes<br>No<br>No
28	Kaliyaperumal et al. 2022 (32)	Semantic modelling of common data elements for rare disease registries, and a prototype workflow for their deployment over registry data	Here we describe the process of data modelling within the EJP RD, as applied to the set of CDEs defined by the EU RD Platform.	FAIR<br>Legal<br>Organisational	Yes<br>No<br>Yes	Yes<br>No<br>No
29	Kinsner et al. 2018 (50)	A sustainable solution for the activities of the European network for surveillance of congenital anomalies: EUROCAT as part of the EU Platform on Rare Diseases Registration	This paper describes the functioning of EUROCAT in the new setting, and gives an overview of the activities and the organisation of the JRC-EUROCAT Central Registry.	FAIR<br>Legal<br>Organisational	No<br>Yes<br>Yes	Yes<br>Yes<br>Yes

N	CITATION	TITLE	OBJECTIVE	D*	C**	R***
30	Klin et al. 2017 (40)	European Reference networks for rare diseases: what is the conceptual framework?	The purpose is to underline the principles which should better be respected to ensure that the ERNs deliver the expected added-value, and finally recommend possible instruments and tools which could promote, nationally and at the European level, the exchange of knowledge and information within and between ERNs, and to support the establishment of collaborative network structures nationally.	FAIR<br>Legal<br>Organisational	No<br>No<br>Yes	No<br>No<br>No
31	Kodra et al. 2018 (56)	Recommendations for Improving the Quality of Rare Disease Registries	We report on a list of recommendations, developed by a group of experts, includingmembers of patient organizations, to be used as a framework for improving the quality of RDregistries. This	FAIR<br>Legal<br>Organisational	No<br>Yes<br>No	Yes<br>Yes<br>Yes
32	Kölker et al. 2022 (35)	Rare Disease Registries Are Key to Evidence-Based Personalized Medicine: Highlighting the European Experience	To illustrate the benefits and limitations of patient registries on rare disease research this review focuses on inherited metabolic diseases.	FAIR<br>Legal<br>Organisational	Yes<br>No<br>Yes	Yes<br>No<br>Yes
33	Kourime et al. 2017 (51)	An assessment of the quality of the I-DSD and the I-CAH registries - international registries for rare conditions affecting sex development	This study was performed to evaluate the I-DSD and I-CAH Registries and identify their strengths and weaknesses.	FAIR<br>Legal<br>Organisational	No<br>No<br>Yes	No<br>Yes<br>Yes
34	Laurie et al. 2022 (24)	The RD-Connect Genome-Phenome Analysis Platform: Accelerating diagnosis, research, and gene discovery for rare diseases	The RD Connect GPAP has been used as the primary analysis tool in a number of large European projects and is involved in many ongoing projects and initiatives. Here, we describe in detail the RD Connect GPAP, a scalable and interoperable online system which facilitates the collation, analysis, interpretation, and sharing of integrated genome phenome datasets, with a particular focus on RD case diagnosis and novel gene discovery.	FAIR<br>Legal<br>Organisational	Yes<br>Yes<br>Yes	Yes<br>Yes<br>Yes
35	Lochmüller et al. 2017 (26)	The International Rare Diseases Research Consortium: Policies and Guidelines to maximize impact	Not explicitly stated	FAIR<br>Legal<br>Organisational	No<br>Yes<br>Yes	Yes<br>Yes<br>No
36	Lochmüller et al. 2018 (78)	RD-Connect, NeurOmics and EURenOmics: collaborative European initiative for rare diseases	In this review, we present the accomplishments of these three projects, their role in the RD research and outlooks.	FAIR<br>Legal<br>Organisational	No<br>No<br>No	Yes<br>Yes<br>Yes

N	CITATION	TITLE	OBJECTIVE	D*	C**	R***
37	Lynn et al. 2017 (75)	How the EUCERD Joint Action supported initiatives on Rare Diseases	In this paper, the authors aim to raise awareness of the work done by the EUCERD Joint Action on behalf of the rare disease community and the policies established.	FAIR<br>Legal<br>Organisational	No<br>Yes<br>No	No<br>No<br>No
38	Maiella et al. 2018 (36)	Harmonising phenomics information for a better interoperability in the rare disease field	The HIPBI-RD project aims to provide the community with an integrated, RD-specific informatics ecosystem that harmonises the way phenomics information is stored in databases and in patient files worldwide, and thereby contributing to interoperability between different sources such as databases, registries and biobanks (both patient centered and gene-centered).	FAIR<br>Legal<br>Organisational	No<br>No<br>Yes	Yes<br>No<br>No
39	Mascalzoni et al. 2013 (73)	Rare diseases and now rare data?	Not explicitly stated	FAIR<br>Legal<br>Organisational	No<br>Yes<br>No	No<br>No<br>No
40	Mascalzoni et al. 2017 (52)	The Role of Solidarity(-ies) in Rare Diseases Research	Not explicitly stated	FAIR<br>Legal<br>Organisational	No<br>No<br>Yes	No<br>No<br>No
41	Mathoulin et al. 2019 (72)	Evidence-based data and rare cancers: The need for a new methodological approach in research and investigation	We describe the barriers in the development of evidence-based medicine and the possibilities of strong development in research and clinical investigations by using rare cancer examples.	FAIR<br>Legal<br>Organisational	No<br>Yes<br>No	No<br>No<br>No
42	McCormack et al. 2016 (53)	"You should at least ask". The expectations, hopes and fears of rare disease patients on large-scale data and biomaterial sharing for genomics research	To this end, this exploratory study documents the hopes, expectations and concerns of RD patients, as identified by participants themselves, in the changing landscape of NGS and international data sharing.	FAIR<br>Legal<br>Organisational	No<br>No<br>Yes	No<br>No<br>No
43	Menesidou et al. 2019 (17)	Specification of consent management and decentralized authorization mechanisms for HR Exchange	This deliverable provides the first version of the specification of consent management and decentralized authorization mechanisms for health records in InteropEHRate.	FAIR<br>Legal<br>Organisational	No<br>Yes<br>No	No<br>No<br>No

N	CITATION	TITLE	OBJECTIVE	D*	C**	R***
44	Monaco et al. 2014 (25)	The challenge for a European network of biobanks for rare diseases taken up by RD-Connect	In this paper, the challenges to be addressed in dealing with RD biobanks will be described, along with the potential solutions envisaged within the newly started RDConnect program funded by the European Commission	FAIR<br>Legal<br>Organisational	No<br>Yes<br>Yes	No<br>Yes<br>Yes
45	Montserrat et al. 2019 (41)	Policies and actions to tackle rare diseases at European level	Not explicitly stated	FAIR<br>Legal<br>Organisational	No<br>No<br>Yes	No<br>No<br>Yes
46	Mora et al. 2015 (37)	The EuroBioBank Network: 10 years of hands-on experience of collaborative, transnational biobanking for rare diseases	This report describes the development of the EBB network over the past decade, its achievements, and the major challenges it has already faced and expects to face in the future.	FAIR<br>Legal<br>Organisational	No<br>Yes<br>Yes	No<br>Yes<br>No
47	Nguyen et al. 2019 (54)	Model consent clauses for rare disease research	The model consent clauses presented in this article have been drafted to highlight consent elements that bear in mind the trends in rare disease research, while providing a tool to help foster harmonization and collaborative efforts.	FAIR<br>Legal<br>Organisational	No<br>Yes<br>Yes	No<br>Yes<br>No
48	Parker et al. 2014 (42)	The pooling of manpower and resources through the establishment of European reference networks and rare disease patient registries is a necessary area of collaboration for rare renal disorders	This review aims to provide guidance on emerging concepts and policy related to European reference networks (ERNs) for rare diseases (RDs) and the development and management of RD patient registries.	FAIR<br>Legal<br>Organisational	Yes<br>Yes<br>Yes	Yes<br>Yes<br>Yes
49	Pejcic et al. 2017 (43)	Transposition and implementation of EU rare disease policy in Eastern Europe	A 10-indicator set was elaborated to structure the review and to describe rare disease activities in 14 Eastern European countries.	FAIR<br>Legal<br>Organisational	No<br>No<br>Yes	No<br>No<br>No
50	Peloquin et al. 2020 (68)	Disruptive and avoidable: GDPR challenges to secondary research uses of data	In this article, we describe challenges that GDPR has posed for biobanks and databanks and for researchers who use those banked resources for secondary research. We discuss the limitations inherent in the few pathways that GDPR makes available for secondary research, given that such pathways rely upon complex and varied laws of individual European Union member states. We advocate mitigation of these difficulties through regulatory guidance in order to allow important scientific research to continue.	FAIR<br>Legal<br>Organisational	No<br>Yes<br>No	No<br>No<br>No

N	CITATION	TITLE	OBJECTIVE	D*	C**	R***
51	Pormeister et al. 2018 (69)	Genetic research and applicable law: the intra-EU conflict of laws as a regulatory challenge to cross-border genetic research	This article aims to analyse the question of applicable national law within the data protection framework specifically in the context of genetic research.	FAIR<br>Legal<br>Organisational	No<br>Yes<br>No	No<br>No<br>No
52	Reza et al. 2017 (55)	MRC Centre Neuromuscular Biobank (Newcastle and London): Supporting and facilitating rare and neuromuscular disease research worldwide	Nine years after the establishment of the MRC Centre Biobank, many high profile research publications have highlighted the positive impact of neuromuscular biobanking for translational research and proven this facility to be a unique repository source for diagnostics, basic science research, industry, drug development, and therapy.	FAIR<br>Legal<br>Organisational	No<br>No<br>Yes	No<br>Yes<br>Yes
53	Santoro et al. 2015 (38)	Rare disease registries classification and characterization: a data mining approach	The objective of this study is to define a classification and characterization of RDRs in order to identify different profiles and informative needs.	FAIR<br>Legal<br>Organisational	Yes<br>No<br>Yes	Yes<br>No<br>Yes
54	Schaaf et al. 2021 (59)	The Registry Data Warehouse in the European Reference Network for Rare Respiratory Diseases - Background, Conception and Implementation	The objectives of this work are to present the aims, a conception and software-implementation of the RDW, as well as an interoperability approach between existing ERN-Lung registries.	FAIR<br>Legal<br>Organisational	Yes<br>No<br>No	Yes<br>No<br>No
55	Schaefer et al. 2020 (10)	The use of machine learning in rare diseases: a scoping review	This scoping review aims to address this gap and explores the use of machine learning in rare diseases, investigating, for example, in which rare diseases machine learning is applied, which types of algorithms and input data are used or which medical applications (e.g., diagnosis, prognosis or treatment) are studied.	FAIR<br>Legal<br>Organisational	No<br>No<br>Yes	No<br>No<br>Yes
56	Schee et al. 2017 (39)	Personalized Medicine: What's in it for Rare Diseases?	Emerging technologies such as Whole Genome Sequencing (WGS), Whole Exome Sequencing (WES) or Low-Coverage Sequencing (LCS) have proven that recent failures in stratified medicine show the need for better understanding of the molecular basis of rare diseases (RDs).	FAIR<br>Legal<br>Organisational	No<br>No<br>Yes	No<br>No<br>Yes
57	Sernadela et al. 2017 (45)	Linked Registries: Connecting Rare Diseases Patient Registries through a Semantic Web Layer	In this work, we have developed a new semantic layer on top of existing patient registries, to allow extract- ing anonymised data from the original datasets, translate them to a common shared exchange model, and make them available to the research community	FAIR<br>Legal<br>Organisational	Yes<br>No<br>Yes	Yes<br>No<br>No

N	CITATION	TITLE	OBJECTIVE	D*	C**	R***
58	Taruscio et al. 2013 (22)	The current situation and needs of rare disease registries in Europe	The present paper reports on the results of an inquiry carried out by EPIRARE on the main activities and needs of existing RD registries in the European Union (EU), the way they deal with methodological, technical and regulatory issues and the way they find resources to carry on their activities. Also, this study is aimed at collecting the opinion of registrars on possible tools and services that may be developed in support to their activities. This will help identify possible options for the implementation of the EPIRARE.	FAIR<br>Legal<br>Organisational	No<br>No<br>Yes	No<br>Yes<br>No
59	Thompson et al. 2014 (12)	RD-Connect: an integrated platform connecting databases, registries, biobanks and clinical bioinformatics for rare disease research	In this review, we provide an overview of the objectives and initial achievements of one of the first projects to be funded under the IRDiRC.	FAIR<br>Legal<br>Organisational	Yes<br>Yes<br>Yes	Yes<br>Yes<br>No
60	Vukovic et al. 2022 (65)	Enablers and barriers to the secondary use of health data in Europe: general data protection regulation perspective	The main objective of this work is to provide insights into the relation between a regulation " GDPR "and data practice " secondary use of health data. It does so by specifically exploring how reearchers working on cross-border projects and exchanging health data see the GDPR, how it affects their work, where they see the GDPR as an enabler and where as a barrier to the cross-border health data exchange.	FAIR<br>Legal<br>Organisational	No<br>Yes<br>No	No<br>No<br>No
61	WHO 2021 (66)	The protection of personal data in health information systems- principles and processes for public health	This guidance document is part of WHO Regional Office for Europe s work on supporting Member States in strengthening their health information systems (HISs). Helping countries to produce solid health intelligence and institutionalized mechanisms for evidence-informed policy-making has traditionally been an important focus of WHOs work and continues to be so under the European Programme of Work 2020 "2025.1	FAIR<br>Legal<br>Organisational	No<br>Yes<br>No	No<br>Yes<br>No
62	Zurek et al. 2021 (80)	Solve-RD: systematic pan-European data sharing and collaborative analysis to solve rare diseases	Not explicitly stated	FAIR<br>Legal<br>Organisational	No<br>No<br>No	Yes<br>Yes<br>Yes

N	CITATION	TITLE	OBJECTIVE	D*	C**	R***
63	Austin et al. 2018	Future of Rare Diseases Research 2017-2027: An IRDiRC Perspective	Given the unusually broad scope of IRDiRC—in science, constituencies, and geography—the IRDiRC goal-setting process incorporated an unusually broad series of criteria.	Any	No<br>No<br>No	Yes
64	Aymé et. al. 2015	Rare diseases in ICD11: making rare diseases visible in health information systems through appropriate coding	Not explicitly stated	Any	No<br>No<br>No	Yes
65	Badowska et al. 2018	RD-Connect, NeurOmics and EURenOmics: collaborative European initiative for rare diseases	In this review, we present the accomplishments of these three projects, their role in the RD research and outlooks.	Any	No<br>No<br>No	Yes
66	Directorate-General for Health and Food Safety 2014	Recommendation on ways to improve codification for rare diseases in health information systems	In the context of the improvement of codification for rare diseases being cited as a priority in the Council Recommendation on an action in the field of rare diseases, the Commission Expert Group on Rare Diseases Adopted the Recommendations.	Any	No<br>No<br>No	Yes
67	European commission 2020	Communication from the commission to the European Parliament, the council, the European economic and Social Committee and the committee of the regions.	This Communication outlines a strategy for policy measures and investments to enable the data economy for the coming five years.	Any	No<br>No<br>No	Yes
68	Evangelista et al. 2016	The context for the thematic grouping of rare diseases to facilitate the establishment of European Reference Networks	In this paper we have focused on the process by which a decision was reached and adopted by the CEGRD as to how we could efficiently group RD in order to support the constitution of well-functioning ERNs.	Any	No<br>No<br>No	Yes
69	Ferrelli et al. 2017	Health Systems Sustainability and Rare Diseases	The paper is addressing aspects of health system sustainability for rare diseases in relation to the current economic crisis and equity concerns. It takes into account the results of the narrative review carried out in the framework of the Joint Action for Rare Diseases (Joint RD-Action) "Promoting Implementation of Recommendations	Any	No<br>No	Yes

N	CITATION	TITLE	OBJECTIVE	D*	C**	R***
			on Policy, Information and Data for Rare Diseases", that identified networks as key factors for health systems sustainability for rare diseases.		No	
70	Foreman et. al. 2022	DECIPHER: Supporting the interpretation and sharing of rare disease phenotype-linked variant data to advance diagnosis and research	To present examples of the genotype/phenotype data deposited and shared with the rare disease community. In addition, we present the tools provided by DECIPHER to assess the pathogenicity of variants according to international standards, and the utility of DECIPHER to map the clinically relevant part of the assayable human genome.	Any	No<br>No<br>No	Yes
71	Hendolin 2021	Towards the European health data space: from diversity to a common framework	The Towards European Health Data Space (TEHDAS) joint action advances more extensive use of health data across Europe. It supports the European Commission's aim in creating a harmonised internal market for health data by providing substance to the European Commission's forthcoming legislative proposal on the Health Data Space.	Any	No<br>No<br>No	Yes
72	Kalyvas et al. 2014	Big Data: A business and legal guide.	Supplies a clear understanding of the interrelationships between Big Data, the new business insights it reveals, and the laws, regulations, and contracting practices that impact the use of the insights and the data. Providing business executives and lawyers (in-house and in private practice) with an accessible primer on Big Data and its business implications, this book will enable readers to quickly grasp the key issues and effectively implement the right solutions to collecting, licensing, handling, and using Big Data.	Any	No<br>No<br>No	Yes
73	Köhler et al. 2019	Expansion of the Human Phenotype Ontology (HPO) knowledge base and resources	The project has added new content, language translations, mappings and computational tooling, as well as integrations with external community data.	Any	No<br>No<br>No	Yes

***D – domains; **C – challenges; ***R – recommendations**
